# Identification and expression of cysteine sulfinate decarboxylase, possible regulation of taurine biosynthesis in *Crassostrea gigas* in response to low salinity

**DOI:** 10.1038/s41598-017-05852-6

**Published:** 2017-07-14

**Authors:** Xuelin Zhao, Qi Li, Qian Meng, Chenyang Yue, Chengxun Xu

**Affiliations:** 1 Key Laboratory of Mariculture, Ministry of Education, Ocean University of China, Qingdao, 266003 China; 2 School of Marine Sciences, Ningbo University, Ningbo, 315211 China; 3 Laboratory for Marine Fisheries Science and Food Production Processes, Qingdao National Laboratory for Marine Science and Technology, Qingdao, China

## Abstract

Taurine has been reported high amounts in marine animals to maintain osmotic balance between osmoformers and sea water. Approximately 80% of the total amino-acid content is taurine in Pacific oyster *Crassostrea gigas*, an intertidal and euryhaline species. In this study, we cloned the two copies of cysteine sulfinate decarboxylase (CSAD), the key enzyme in taurine biosynthesis pathway, screened in oyster genome data. Sequentially, we compared the expression patterns of CgCSAD1 and CgCSAD2 under low salinity treatment (8‰ and 15‰) using different families from two populations. There was no correlation between the expression of CSAD and the different population. Notably, CgCSAD1 increased significantly in treated groups for 24 h, but CgCSAD2 had no significant differentiation. Moreover, the results of CgCSAD1 interference provided the evidence of the positive correlation between CgCSAD1 expressions and taurine contents. The zinc finger domain showed in multi-alignment results may be the important character of CgCSAD1 as the key enzyme in taurine biosynthesis to regulate taurine pool in response to low salinity. This study provides a new evidence for the important role of taurine in adaptation to low salinity in oyster. In addition, it is a good model to discuss the function and evolution of the duplication in mollusks.

## Introduction

Environmental factors, especially salinity, in intertidal zones fluctuated acutely owing to tides, rainfall, surface runoff and so on. Osmoconforming marine animals can result in large changes in the osmotic concentration of hemolymph in order to adapt to fluctuations in salinity of ambient seawater^1, 2^. One of the primary strategies of osmoconforming animals to keep the osmotic balance is the manipulation of intracellular levels of organic osmolytes, especially free amino acids^3^. For marine molluscs, as osmoconforming animals, only a few free amino acids predominantly contribute to the intracellular pool, such as alanine, glycine, proline, taurine and glutamate. The content of taurine is lower in freshwater species due to the minimized osmotic equilibrium between the cells of them and the respective extracellular fluids^4^. Unlike freshwater invertebrates, the cells and extracellular fluids are very close to sea water in osmotic concentration, and their intracellular inorganic ion composition is only slightly higher than freshwater species. So free amino acids, as organic osmolytes, maintain osmotic balance between extracellular and intracellular fluids in marine species^5^.

The Pacific oyster *Crassostrea gigas* is a euryhaline bivalve which can experience rapid and dramatic salinity fluctuations from below 10‰ to above 35‰^6^. As was reported, the osmolality of their hemolymph could change from 800 to 380 mOsm/kg within eight hours with the changes of seawater^7^. The decrease in osmolality and increase in water content of the mantle within 8 h after the exposure to hypo-osmolality were caused by the permeation of water into the oyster. Thereafter the water content decreased gradually up to 72 h, while the osmolality did not change^7^. There is no evidence of ionic regulation for Na^+^, Ca^2+^ and Mg^2+^ in *C. gigas* ^8^. Hopkins^9^ gave 33 to 41% seawater as the lower limit for effective pumping in the Pacific oyster. Interestingly, taurine, as the primary osmolyte accounts for approximately 80% of the total amino-acid content in the Pacific oyster^10^ and were significantly influenced the pattern of the change in total content of FAA in adaptation to hypo-osmolality^7^.

Taurine, 2-aminoethanesulfonic acid, is a unique sulfur-containing free amino acid because it does not form peptide bonds with other amino acids^11^. As an important osmolyte, taurine was found to participate in osmoregulation in many invertebrates, fishes and mammals^12^. There are multiple biosynthetic pathways for taurine synthesis in animals^11^. Marine bivalves mainly use cysteine as raw material to maintain taurine pool^3^. There are two pathways synthesized from cysteine, which have been identified in mammals^13, 14^. One way includes the oxidation of cysteine to cysteine sulfinic acid (CSA) by cysteine dioxygenase (CDO), and decarboxylation to hypotaurine by cysteine sulfinate decarboxylase (CSAD). In this pathway, CSAD has been characterized as the key enzyme that determines taurine biosynthesis capability^15, 16^. The other is cysteamine pathway, converting cysteamine to hypotaurine by cysteamine dioxygenase (ADO). The oxidation of hypotaurine to taurine has no enzymes reported, which is thought to be a spontaneous process^12, 15^.

Besides the endogenous biosynthesis of taurine, the transport of taurine by taurine transporter (TauT) is another way to control taurine content for osmotic balance. Studies on TauT gene expression and protein expression in low or high salinity have been conducted in oysters^17^ and mussels^10, 18^. However, the key enzymes of taurine metabolism in Mollusca is have rarely been reported^19^. Although hypotaurine and taurine are abundant in mollusc, the biosynthesis pathway remains unclear.

The annotation information of genome and transcriptome of *C. gigas* provides the sequences of relevant enzymes^20, 21^. In our previous study^22^, we found taurine might be the main osmolyte for isotonicity at low salinity. Meanwhile, the differential expression of CSAD was reported in oyster transcriptome in response to low salinity^21, 23^. In addition, there are two copies of CSAD genes in *C. gigas* and the change in expression of these two copies displayed differentiation in transcriptomic data. Therefore, in this study, we tried to identify genes involved in taurine synthesis in the gill tissue of *C. gigas*. We cloned the two copies of CSAD genes searched from the oyster genome, and compared the spatial and temporal expressions of the two genes in different tissues and different salinity treatments. Subsequently, we silenced the CgCSAD1 to estimate the correlation between the expression of CSAD and taurine contents in gill tissues by RNA interference. On the basis of the results of these analysis, we discussed the roles of CSAD genes and other related genes in taurine biosynthesis and mollusc osmoregulation.

## Materials and Methods

### Samples collection and mating design

C*. gigas* was obtained from two different wild populations Weihai (salinity 30) and Dongying (salinity 16) in 2015. The two sites were intended to represent two oyster-growing environments in Shandong Province. Diallel cross was performed between the two wild populations in June, including two hybrid families DW1 (D♀ × W♂), DW2 (D♂ × W♀) and four parental families WW1 (W♀ × W♂), WW2 (W♀ × W♂), DD1 (D♀ × D♂) and DD2 (D♀ × D♂). After six months’ growing in natural water in Rushan, China, the juvenile oysters were moved to the laboratory (Key laboratory of mariculture of ministry of education, Qingdao) for experiments.

### Salinity stress experiment

The Families WW1, WW2, DD1, DD2, DW1 and DW2 were acclimatized for two weeks in 30‰ filtered seawater at 18 °C in our laboratory. After that, each family was separated in tanks filled with 8‰, 15‰, and 30‰ filtered seawater, respectively. There were at least 20 individuals of each family in each treatment. After 8 hours and 24 hours’ treatments, the gill tissues of oysters in each tank were dissected and saved in RNA store (Dongsheng Biotech) at −80 °C, respectively.

### Tissue dissection

Adult Pacific oysters (shell length: 13–15 cm) were purchased from a commercial oyster farm in Weihai, Shangdong Province, China. Gill, mantle, adductor muscle, labial palp, digective gland, gonad and hemolymph were dissected after acclimation. Hemolymph was frozen immediately using liquid nitrogen, and other tissues were saved in RNA store. All the tissues were stored at −80 °C until use.

### RNA extraction

Total RNA of the tissues were extracted using TRIzol (Life Technologies, Carlsbad, CA) according to the manufacturer’s instructions. RNA integrity was checked by agarose gel electrophoresis with the proportion of the ribosomal bands as a ratio of 2.0^8^.

### Molecular cloning and characterization of full length cDNAs

Whole coding regions of CDO and CSAD cDNA were cloned utilizing the partial cDNA sequences from the genome of *C. gigas*. First strand cDNA was produced by SMART RACE cDNA Amplification Kit (Clontech) and used as template for 3′ or 5′ rapid amplification of cDNA ends (RACE) PCR. Specific primers were further designed for RACE PCR with Primer Premier 5 software to obtain upstream and downstream sequences. PCR was performed for 35 cycles at 94 °C for 30 s, 60 °C for 30 s and 70 °C for 2 min. All the purified PCR products were gel purified and ligated into pEASY-T1 vector (Transgen Biotech). The positive clones were sequenced in Sangon Biotech CO., Ltd (Shanghai). All the primers used in this study were listed in Table 1.

**Table 1:** The primer used in clone and RNA interference.

Gene name	Primer name	Primer sequence (5′–3′)	Primer Introduction
CgCSAD1	T7-26339f	GATCACTAATACGACTCACTATAGGGACAGTTTGGGAGGATGTTGGTT	Prepare the template for amplification of dsRNA
CgCSAD1	T7-26339r	GATCACTAATACGACTCACTATAGGGACTGAATGGACTTGTCGCCG	Prepare the template for amplification of dsRNA
CgCSAD1	26339HEf	CACAAAAGAATCCACGAC	partial coding region of CgCSAD1 gene
CgCSAD1	26339HEr	TCATTCCCTCCTTGACCT	partial coding region of CgCSAD1 gene
CgCSAD1	26339-3′-628	ATGTTCTGCCCTGGCGGTTCTATT	3′RACE PCR for CgCSAD1
CgCSAD1	26339-3′-1529	CAGCCTCAATGATGGTCGGTTAC	3′RACE PCR for CgCSAD1
CgCSAD1	26339-5′-853	GAATCATCTTTCCATTAGCATCAGTCT	5′RACE PCR for CgCSAD1
CgCSAD1	26339-5′-452	GGATGGGCAACTTTCACGCTGTA	5′RACE PCR for CgCSAD1
CgCSAD2	22071HEf	CATCCCAAAGAGTTAGAGG	partial coding region of CgCSAD2 gene
CgCSAD2	22071HEr	GAAGAAATTGACATGCTCC	partial coding region of CgCSAD2 gene
CgCSAD2	22071-3′-319	GCCTGGCTCACTGAGGTCCTGAATA	3′RACE PCR for CgCSAD2
CgCSAD2	22071-3′-159	GGGAGAGACTGGGAAAGGTGGCT	3′RACE PCR for CgCSAD2
CgCSAD2	22071-5′-866	CGGTATTTCTTGGACAACAAGG	5′RACE PCR for CgCSAD2
CgCSAD2	22071-5′-312	CAACCTGTAATCATCCAGTCCACT	5′RACE PCR for CgCSAD2

### Long double-stranded RNAs (dsRNAs) preparation

Double-stranded RNAs (dsRNAs) specific to *C. gigas* CSAD-1 were synthesized *in vitro* using T7 Quick High Yield RNA Synthesis Kit (New England Biolabs) according to manufacturer’s instructions. Briefly, a partial cDNA fragment of CSAD-1 (765 bp) was PCR-amplified using a pair of primers, T7-26339f and T7-26339r listed in Table 1. The amplified fragments were purified and used to amplify the dsRNA as the template. Digestion of the template DNA remaining in the dsRNA, and purification of dsRNA were conducted using the reagents in the kit.

### Oysters and dsRNA administration

Pacific oysters (shell length: 13–15 cm) purchased from a commercial oyster farm in Weihai, Shangdong Province, China were used for RNAi experiments and kept in filtered seawater at 18 °C for two weeks. For the experiment, oysters were divided randomly into two groups. One group was injected in 100 μl, 1 μg/μl dsRNA, and other group was injected in 100 μl, 150 mM NaCl as control group. After 48 h post-injection, each group was separated in 3 sub-groups and cultured in 8‰, 15‰, and 30‰ filtered seawater, respectively. The gill tissues of oysters were dissected and divided into two groups. One was saved in RNA store at −80 °C for RNA extraction, the other was frozen immediately using liquid nitrogen and stored at −80 °C for taurine extraction.

### Molecular phylogenetic analyses

Alignment of the sequences and construction of neighbor-joining tree with 1,000 bootstrap replications were performed using the MEGA software program v5^24^. The tree was rooted by evolution-related enzymes, HDC (histidine decarboxylase) of human.

### Reverse-transcription PCR

The cDNA for qRT-PCR was prepared using PrimeScript 1^st^ Strand cDNA Synthesis Kit (TaKaRa). Firstly, 10 μl reaction consisted of 2 μl 5 × gDNA Ereaser Buffer, 1 μl gDNA Eraser, 2 μl total RNA and 5.0 μl RNase-free water under 42 °C for 2 min to eliminate genome DNA. Then, 1 μl PrimeScript RT Enzyme Mix I, 1 μl RT Primer Mix, 4.0 μl 5 × PrimerScript Buffer 2 and 4.0 μl RNase-free water were added to the above 10 μl reaction. The reverse-transcription PCR program was set with 37 °C for 15 min and 85 °C for 5 s.

### Quantitative real-time PCR analysis

In order to understand the spatio-temporal expression of CgCSAD1 and CgCSAD2 and compare the mRNA levels of the related genes in the taurine metabolism pathway in different salinity conditions and RNAi experiments, quantitative real-time PCR (qRT-PCR) was conducted for relative quantification of mRNA using SYBR Green real-time PCR kit (Takara) with Roche Lightcycler 480 (Roche, CA). The qRT-PCR primers were designed with Primer Premier 5 software, primers with amplification efficiency 95–105% by means of standard curve drawn using dilution series. No template controls (NTC) were present for all genes (primer pairs). The 20 μl PCR mixture contained 10 μl of SYBR Premix Ex Taq II (TaKaRa), 0.8 μl of 10 μM forward primer, 0.8 μl of 10 μM reverse primer, 2 μl of cDNA template and 6.4 μl of DEPC treated water. The qRT-PCR cycling conditions were 95 °C for 5 s, 60 °C for 30 s and 72 °C for 30 s. At the end of the PCR cycles, melting curve analyses were performed. Elongation factor 1 α was used as an internal control, which was used as internal reference gene of qRT-PCR for salinity treatments in the Pacific oyster^21^. Six samples were used in each group to reduce experimental error. The primers were listed in Table 2.

**Table 2:** The primer used in qPCR.

Gene name	Abbreviation	Accession number	Primer sequence (5′–3′)
cysteine sulfinic acid decarboxylase	CgCSAD1	KY418036	F: TCTGCCAATGCCTCGTACCT, R: CGTTCATCTCCCTTGTTCTTCC
cysteine sulfinic acid decarboxylase	CgCSAD2	KY418037	F: AAGCACCATGTTGATCGGG, R: TCCAAGCCTGTCAATCTCGT
cysteine dioxygenase	CDO	CGI_10008307	F: ACCACGCAAACGCTCACT, R: TTCATTTCGCCGTCATCC
cysteamine dioxygenase	ADO	CGI_10016854	F: AGGTCAATAGTTTCAGCCAAGCAT, R: AGAACACAGCATTCATCTTCCTCC
glutathione hydrolase	GGT	CGI_10027341	F: GGAGATGGCGATTATGAACC, R: TTGGTATAGTAGTCTCTAGGATGCG
Elongation Factor 1 α	EF	AB122066	F: CAAGAACGGAGATGCTGGTATGG, R: TTTCACTCTTTCCACCGGCTTT

All data was analyzed using 2^−ΔΔCt^ method and the values obtained represented the n-fold difference relative to the control (untreated samples). The data were presented as the relative expression levels (means ± SD, n = 6), and all experimental data were subjected to Student’s *t* test between treated groups and control groups. Significant differences between the treated and corresponding control groups at each time point were indicated as *P < 0.05. The error bars in the graphs represent standard deviations. One-way ANOVA was used to test the changes of gene expression with factor family. We considered differences to be statistically significant at P < 0.05.

### Measurement of taurine by high performance liquid chromatography

The taurine concentration of samples in RNAi experiments was measured with high performance liquid chromatography (HPLC), HP 1100 HPLC system (Agilent Germany). The gill tissues were lypophilized to powder in freezer dryer. The 0.1 g tissue was homogenized in 25 ml sterile water. Then 3 ml solution was mingled with 3 ml 60 g/L sulfosalicylic acid (SSA) and centrifuged for 15 min to pellet precipitated protein. The supernatant was obtained and stored at −80 °C until analysis. Samples were pre-column derivatized with o-phthaladehyde (OPA)/2-mercaptoethanol and separated with a Zorbax Eclipse C18 column (Agilent, Germany), using a gradient elution^25^.

We applied one-way ANOVA to test the changes of taurine levels between RNAi groups and control groups, and different salinity treated groups. We considered differences to be statistically significant at P < 0.05.

## Results

### cDNA cloning and phylogenetic analyses

**Figure 1:**
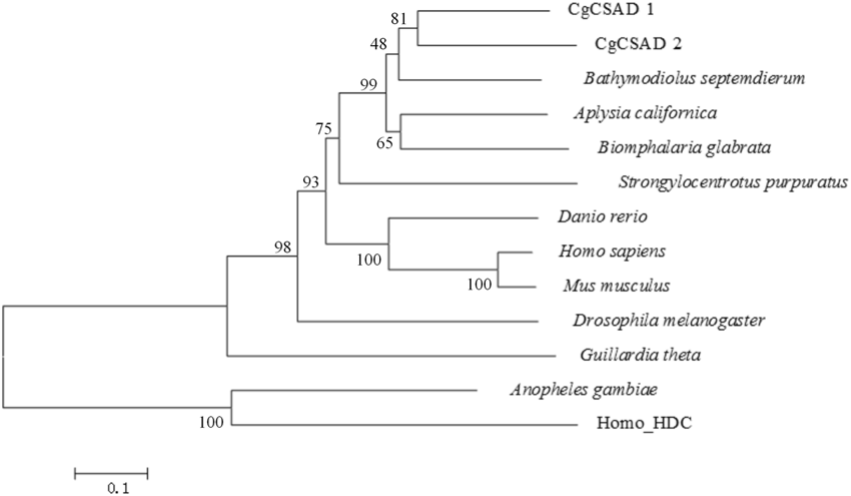
Neighbour-joining tree of full-length CSAD proteins. All the sequences were obtained through the GenBank database.

Two copies of CSAD were cloned in *C. gigas* and were named as CgCSAD1 and CgCSAD2. The CgCSAD1 cDNA was 2,097 bp in length and contained a 60 bp 5′-untranslated region (UTR), a 369 bp 3′-UTR, and a 1,668 bp open reading frame (ORF). It encodes a predicted polypeptide consisting of 555 amino acid residues and an estimated molecular mass of 63.35 kDa; The CgCSAD2 cDNA was 1,713 bp in length and included a 82 bp 5′-UTR, a 146 bp 3′-UTR, and a 1485 bp ORF. It encodes a predicted polypeptide consisting of 494 amino acid residues and an estimated molecular mass of 56.11 kDa. The two copies have no signal peptide. The molecular structure of the two copies contains pyridoxal phosphate (PLP)-dependent amino acid carboxylases domain. Interestingly, a zinc finger domain was in the C-terminus of the protein of CgCSAD1 by PFAM domain prediction (Figure S1). The identity with CSADs of other vertebrates and invertebrates was 50.2% showed similarity (Fig. 1). Within the clade, vertebrate CSADs and mollusk CSADs clustered independently with significant bootstrap support. The full sequences of the two copies were submitted to GenBank under accession numbers, KY418036 and KY418037, respectively.

### Effects of low salinity exposure

**Figure 2:**
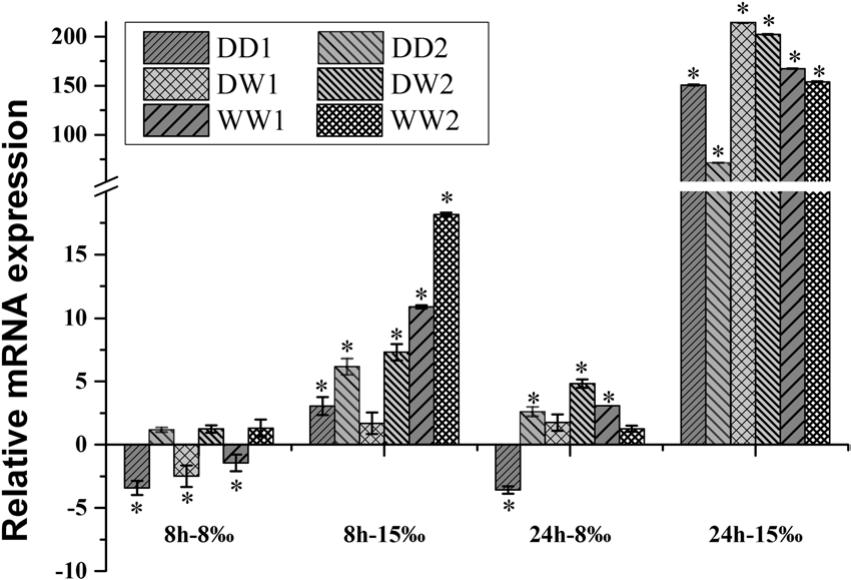
Relative mRNA expression of CgCSAD1 in different families of juvenile oyster under low osmotic stress. Elongation factor 1 α (EF) gene expression was used as internal control. The samples acclimated in 30‰ for 8 hours and 24 hours are the reference samples, respectively. The y-axis represent the fold changes between the expressions of treated samples and reference samples. Vertical bars represent the mean ± SD (N = 6). *Indicated the significant different (fold change > 2, p < 0.05), CSAD: Cysteine sulfinate decarboxylase.

**Figure 3:**
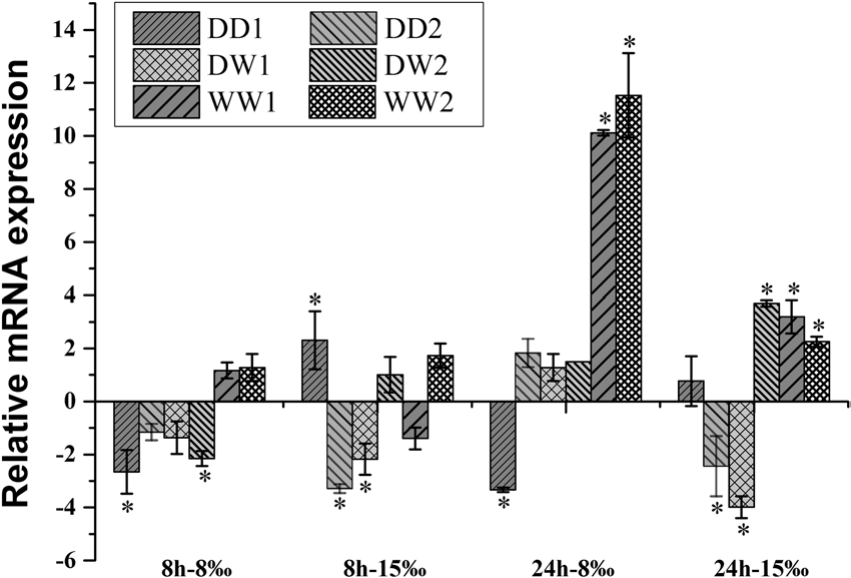
Relative mRNA expression of CgCSAD2 in different families of juvenile oyster under low osmotic stress. Elongation factor (EF) gene expression was used as internal control. The samples acclimated in 30‰ for 8 hours and 24 hours are the reference samples, respectively. The y-axis represent the fold changes between the expressions of treated samples and reference samples. Vertical bars represent the mean ± SD (N = 6). *Indicated the significant different (fold change > 2, p < 0.05), CSAD: Cysteine sulfinate decarboxylase.

With juvenile oysters of WW, DD and DW as samples, the juvenile oysters were cultured in 8‰, 15‰, and 30‰, respectively. After eight and twenty-four hours, gill tissues of six oysters were selected randomly in each treatment. The relative mRNA levels of the CgCSAD1 and CgCSAD2 genes were shown in Figs. 2 and 3, respectively.

Based on the one-way ANOVA results with factor family, the expression of CgCSAD1 was not significantly different among six families under the same treatment (P > 0.1). Cultured in 15‰ condition for eight hours, the expression of CgCSAD1 was up-regulated significantly in five families. After a 24 h treatment, there was no significantly different expression in these families under 8‰. However, the expression of CgCSAD1 was up-regulated hundreds of times under 15‰ for 24 h in all the families.

There was no regular changes in the expression of CgCSAD2 gene between these six families treated in 15‰ for either 8 h or 24 h. However, under 8‰ treatments for 8 h, the expressions in WW families had difference with those in DD families (P = 0.061) and DW families (P = 0.069). Under 8‰ treatments for 24 h, the expression of CgCSAD2 was up-regulated in families WW1 and WW2, while other partial families showed down-regulated (P = 0.039 with DD families and P = 0.068 with DW families).

### Tissue specificity of mRNA expression

**Figure 4:**
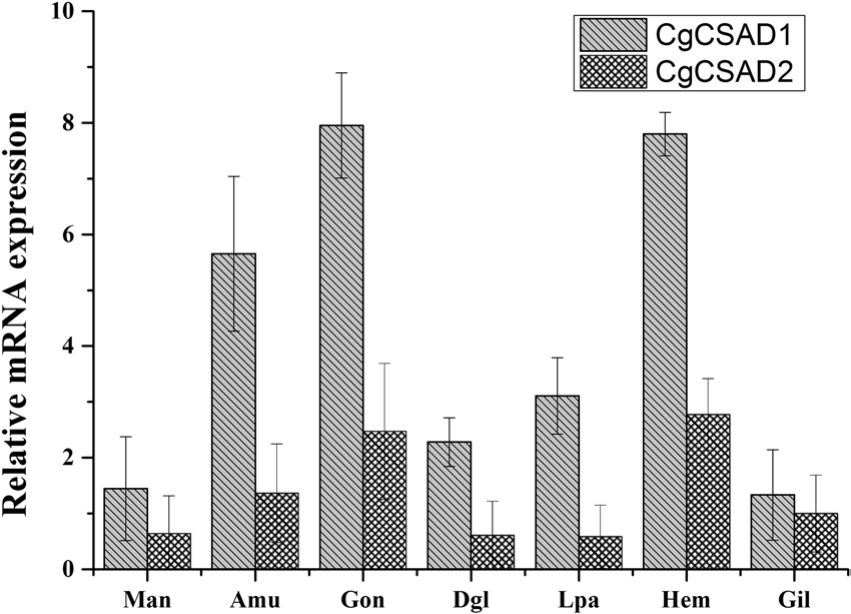
Quantitative real-time PCR analysis of the CgCSAD1and CgCSAD2 gene expression in various tissues. Elongation factor 1 α (EF) gene expression was used as internal control. The y-axis represent the fold changes between the CgCSAD1 and CgCSAD2 expressions of tissues and the CgCSAD2 expression of gill. Vertical bars represent the mean ± SD (n = 6), CSAD: Cysteine sulfinate decarboxylase, Man: mantle, Amu: adductor muscle, Gon: gonad, Dgl: digestive gland, Lpa: labial palp, Hem: hemolymph, Gil: gill.

The transcripts of CgCSAD1 and CgCSAD2 genes were detected in all tissues examined (Fig. 4). The transcript level of CgCSAD1 was higher than that of CgCSAD2 in all tissues. As for CgCSAD1 gene, the transcript level was higher in the gonad, followed by the hemolymph and adductor muscle. Gill and mantle had lower expression of CgCSAD1. The transcript expression of CgCSAD2 was higher in hemolymph and gonad and lower in digestive gland and labial palp.

### Effect on the expression of genes in taurine metabolism after CgCSAD1 interference

**Figure 5:**
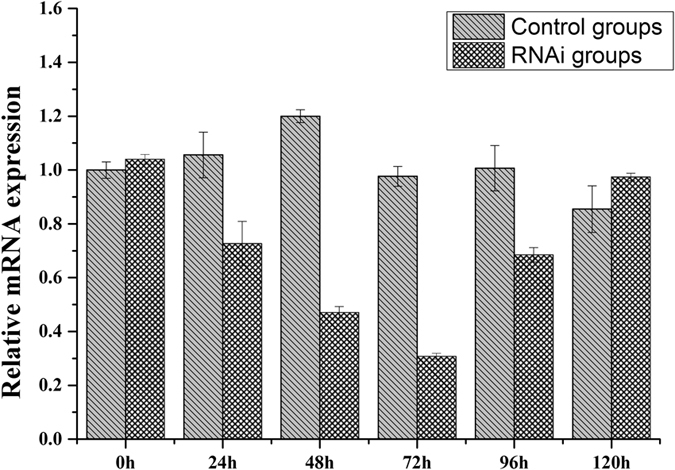
Relative mRNA expression of CgCSAD1 after dsRNA injection. The 0 h of control group are the reference samples. The y-axis represent the fold changes between the expressions of treated samples and reference samples. Vertical bars represent the mean ± SD (N = 6).

Based on the result of pre-experiment (Fig. 5), the expression of CgCSAD1 began to decrease at 24 h after dsRNA injection and it was maintained at a relatively low level from 48 to 72 h. However, oysters injected with 150 mM NaCl did not display significant decreases in CgCSAD1 expression after injection.

**Figure 6:**
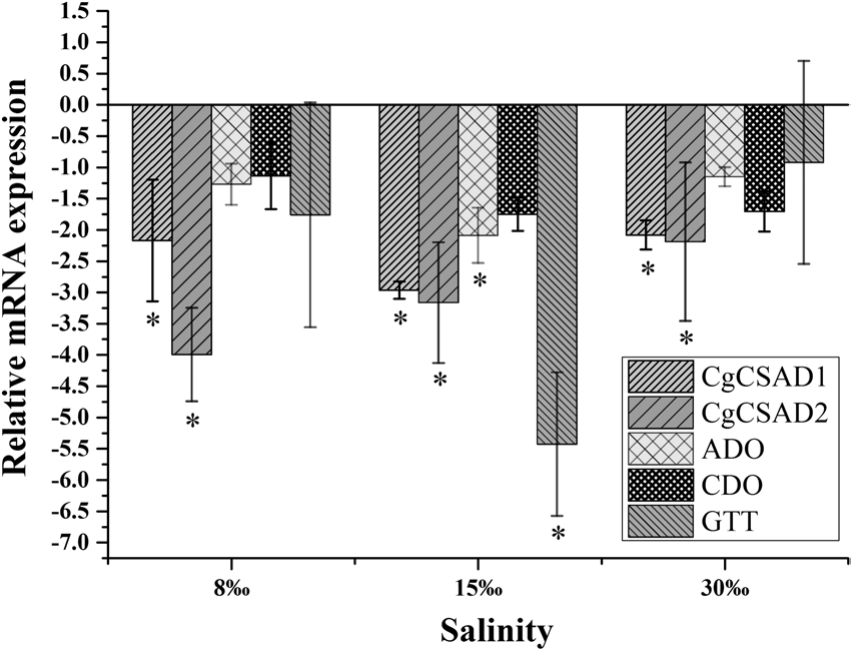
Relative mRNA expression of genes after RNAi of CgCSAD1. Elongation factor 1 α (EF) gene expression was used as internal control. The samples injected in 150 mM NaCl solution and acclimated in 8‰, 15‰ and 30‰ are the reference samples, respectively. Vertical bars represent the mean ± SD (N = 6). *Indicated the significant different (fold change > 2, p < 0.05). CSAD: Cysteine sulfinate decarboxylase, ADO: cysteamine dioxygenase; CDO: cysteine dioxygenase; GTT: glutathione hydrolase.

We tested the effect of CgCSAD1 interference on expression of related genes participating in taurine metabolism pathway, including CgCSAD2, CDO, ADO and GTT. Moreover, after 48 h of dsRNA injection, the oysters injected with dsRNA and 150 mM NaCl were divided into three groups, respectively. Three groups of treatment and control were cultured in 8‰, 15‰ and 30‰ for 24 h, respectively, and the expressions of related genes were detected via real-time quantitative PCR analysis. As the results shown (Fig. 6), the expression of CgCSAD1 decreased significantly after 72 h of dsRNA injection in different salinities. Meanwhile, the transcript level of CgCSAD2 decreased significantly as well. Under 15‰ condition, the expression of ADO and GTT decreased significantly and others had no significant change.

**Figure 7:**
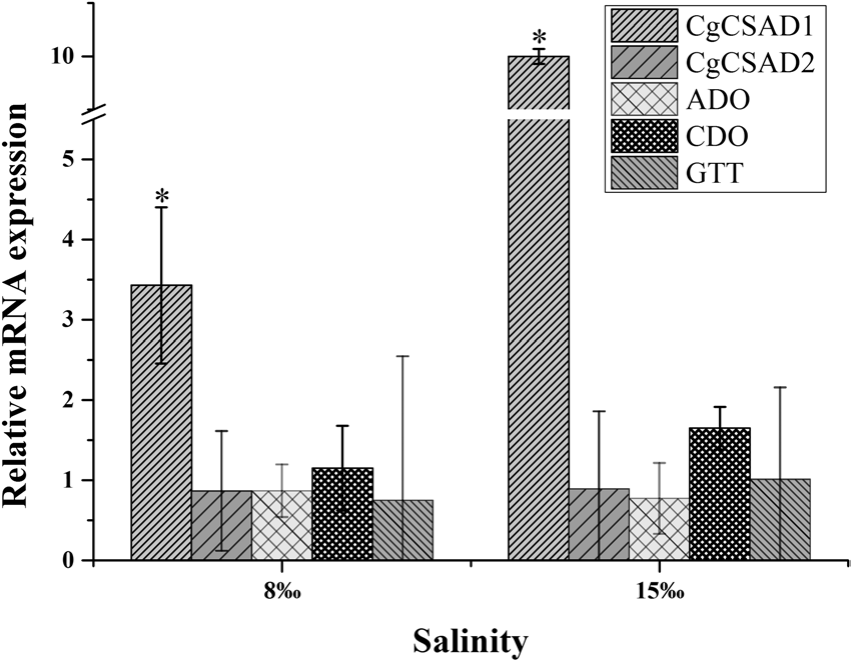
Relative mRNA expression of genes of samples in different salinity after dsRNA injection. Elongation factor 1 α (EF) gene expression was used as internal control. The samples injected with dsRNA of CgCSAD1 and acclimated in 30‰ are the reference samples. The y-axis represent the fold changes between the expressions of treated samples and reference samples. Vertical bars represent the mean ± SD (N = 6). *Indicated the significant different (fold change > 2, p < 0.05). CSAD: Cysteine sulfinate decarboxylase, ADO: cysteamine dioxygenase; CDO: cysteine dioxygenase; GTT: glutathione hydrolase.

Using the CgCSAD1 interference group cultured in 30‰ filtered seawater as control, we compared the expression of related genes in those groups cultured in 8‰ and 15‰ conditions. It showed the expression of CgCSAD1 increased significantly, especially 10 fold in 15‰ filtered seawater, and other genes had no significant change (Fig. 7).

### Effect of CgCSAD1 suppression on taurine contents

CSAD gene is the key enzyme in the pathway of taurine biosynthesis. We measured the changes in taurine content after CgCSAD1 interference to acquire the relationship between the expression of CSAD and taurine contents under different salinity conditions. As Table 3 showed, compared with control groups, taurine contents decreased in groups of CgCSAD1 interference. In different salinity treatments, taurine content was significantly highest in 15‰. Combined the expressions of CSAD, the significantly different expression of CgCSAD1 was in accordance with the significant change of taurine contents in CgCSAD1 interference groups and control groups injected with 150 mM NaCl under salinity 15‰ (Table 3), but the expression of CgCSAD2 had no significant change under different salinity treatments.

**Table 3:** The content of taurine in gill tissues in different treatments.

Group Name	Taurine contents (mg/g dry weight) Average ± SD (n = 6)
8‰T	55.37 ± 2.42^b^
15‰T	60.50 ± 3.11^a^
30‰T	56.06 ± 3.65^bc^
8‰C	60.44 ± 2.80^c^
15‰C	65.57 ± 0.86^b^
30‰C	61.25 ± 4.44^c^

T: CgCSAD1 interference group. C: 150 mM NaCl injected group. Different superscript letters indicate significant difference among means (p < 0.05). Method: one-way ANOVA.

## Discussion

Terrestrial animals or freshwater invertebrates usually seemed to use inorganic ions as osmotic solute. While the bloods of marine invertebrates are very close to sea water in osmotic concentration and much higher than terrestrial animals or freshwater invertebrates in concentration. However, the intracellular osmotic concentration in marine invertebrates is only slightly higher than vertebrate levels. Therefore, free amino acids are utilized as osmotic solute to adapt to changes of environmental salinity^4^. Taurine accounts for 60–80 percent of all free amino acids^26^, as the main amino acid in osmoregulation. In the salinity tolerance experiments in oyster for seven days, the content of taurine changed with the variation of salinity of seawater^21^. It showed that taurine might play a key role in osmoregulation in oyster.

In taurine metabolism pathway, the key enzyme, CSAD has two copies in the genome of *C. gigas*. In this study, we cloned and reported the whole length of the cDNA of the two copies. In bivalve, CSAD genes had been cloned just in deep-sea mussel and only found one copy in its genome^19^. CSAD belongs to the type II PLP-dependent amino acid decarboxylase carboxylase family^27^. We confirmed CgCSAD1 and CgCSAD2 were also members of this family by InterProScan.

In phylogenetic analysis, the two copies of CgCSAD clustered with mussel CSAD, and other two species of mollusks clustered together. Vertebrates and mollusks clustered independently with significant bootstrap support. However, the clustering patterns within the clade were not consistent with phylogeny of animals exactly. In the clade, the mollusks CSAD clustered with the sea urchin CSAD, and did not cluster with insect CSAD, which is also reported in deep-sea mussel. This result may be attributed to the restriction of amino acid substitutions that is common in functional sequences but also to unique diversification in each animal lineage^19^. On the other hand, the natural selection of CSAD gene may rely on habitat, because mollusks and sea urchins live in the sea and insects live on the land.

There is a zinc finger domain in the C-terminus of the protein of CgCSAD1 by PFAM domain prediction (Figure S1) compared with CgCSAD2 and CSAD in the other molluscs and vertebrate. This result was reported in previous study and speculated that this domain may be a transcriptional regulatory site, or an action site sensing the signal transduction of osmotic pressure in response to salinity fluctuation^21^.

CgCSAD1 and CgCSAD2 gene expressions were observed in all the tissues examined. Gonad, haemolymph and adductor muscle exhibited higher levels of expression of the two genes, but gill had a lower expression. It is reported that diets supplemented with taurine could improve the muscle growth of rainbow trout^28^. It may have a similar function in oyster, so that CSAD maintains a high expression in gonad and adductor muscle. Haemolymph is an important place to maintain osmotic balance between extracellular region and external environment. To meet the demand, the oysters may express CSAD at high levels in the haemolymph. To test the correlation between the key enzyme of taurine synthesis pathways and the low salinity adaptation of different oyster population, we cultured one hybrid family and two parental families using two populations and exposed them to the 8‰, 15‰ and 30‰ filtered seawater. The selection of experimental salinity was based on the reported researches. It deduced that 5‰ may exceed the range of oyster salinity tolerance using transcriptomic data by bioinformatics analysis^20, 22^. 8‰ was the lowest known tolerant salinity for *C. gigas* ^29^. It is confirmed that 15‰ was the low salinity to cause stronger response in taurine^22^. However, based on the results of expression of CSAD under the same treated condition, there was no significant differentiation in CgCSAD1 gene between the oysters from two different salinity environments. CgCSAD2 had significant different expression in the WW families under 8‰ treatment compared with the two other families. It may indicate that the ability of taurine regulation is not the main difference between the two oyster populations. However, CgCSAD2 is population-specific in response to low salinity, suggesting it might be correlated with natural selection.

The results of different salinity treatments and timepoints showed that 8‰ was an extreme changes of environmental salinity with weaker expression of CgCSAD1. The expression of CgCSAD1 increased to more than one hundred folds under 15‰ for 24 h. In addition, there were no significant expression after 8 h compared with 24 h. It was reported that the water content of the mantle increased in the first 8 h in response to hypo-osmolality. After that, the water content decreased gradually without the changes of osmolality^7^. Therefore, the taurine regulation might start after 8 h of exposure to hypo-osmolality. Compared with CgCSAD1, the expression pattern of CgCSAD2 was totally different and the expression variation was small in response to low salinity. It was the probable functional difference between CgCSAD1 and CgCSAD2.

To make the function of CgCSAD1 and CgCSAD2 clear, we conducted RNA interference experiment for the correlation between the expression of CSAD and taurine contents. RNA interference is an effective way to induce a post-transcriptional homologous gene-silencing by dsRNA in oyster^30, 31^. dsRNAs in cells are cleaved into small fragments (about 21–23 bp) by an RNase III, and incorporated with RNase into RNA-induced silencing complex (RSIC), which can degrade target mRNA to inhibit the expression of the target gene^32, 33^. In the genome of *C. gigas*, we found there are genes annotated as CDO, ADO and glutathione hydrolase (GGT), which were enzymes participating in taurine and hypotaurine metabolism pathway (map 00430). CDO and GGT were at upstream and downstream of CSAD in the pathway, respectively. ADO was the key enzyme in the side pathway of taurine synthesis. Since CgCSAD2 decreased significantly with CgCSAD1 in CgCSAD1 interference experiment, it is likely that the conserved domain of the two genes resulted in the lightly interference of CgCSAD2. At 15‰, the expressions of ADO were down-regulated significantly, which suggested the side pathway of taurine synthesis did not complement taurine in response to salinity 15‰ when CSAD was silencing. This conclusion was drawn by data *in silico*, too^22^. GTT took part in the taurine catabolism, and was significantly down-regulated with the decreasing of the taurine at 15‰. As showed in Fig. 6, all the gene had the trend of down expression with interference of CgCSAD1, evidencing the key roles of CgCSAD1 in taurine metabolism. Compared even in CgCSAD1 interference groups under different salinities, the expression of CgCSAD1 increased significantly at 8‰ and 15‰ compared with 30‰. It indicated that CgCSAD1 was closely correlated with hypoosmotic stress.

Based on the results of ANOVA analysis on taurine content, the taurine contents were significantly decreasing after the interference of CgCSAD1 under different salinity. It showed there was a positive correlation between the expression of CgCSAD and taurine contents in gill tissues. In addition, taurine contents of samples under 15‰ were significantly different with those under 8‰ and 30‰ conditions either RNAi groups or control groups. It indicated that the taurine contents were correlated with the expression of CgCSAD1 in response to low salinity stress. In addition to taurine biosynthesis pathways for taurine regulation, there was taurine transporter to uptake taurine into body. Taurine transporter required cotransport of amino acids with Na^+^ in integumental tissues^34^. It is reported that acute exposure of isolated gill tissue to 60% artificial sea water resulted in a greater than 85% inhibition of taurine uptake in mussel, but the apparent accumulation of taurine just reduced by approximately 10%^35^. Therefore, we speculated that oysters rapidly swell with the efflux of inorganic ions and organic osmolytes and the influx of water at the start produced by the hypoosmotic stress. Sequentially, the cell will at least partially recover the original volume with time. With the complement of taurine in cells was from the taurine biosynthesis pathways to help the cell volume recovery. The adaptive range of salinity may be determined by the ability of taurine biosynthesis *in vivo*. However, the taurine contents did not showed hundreds of changes in response to salinity 15 with the up-regulation of CgCSAD1. There might be no significant change at functional levels, such as protein content, enzyme capacities and metabolite concentration, at the same time points. In addition, the oxidation of hypotaurine to taurine remained unknown. This process might be one of the reasons resulting in the different changes between gene expression and taurine contents. The further studies need to increase more time points and complement data at functional levels. This study indicated that CgCSAD1 may be an example of gene expanding for salinity adaptation.

However, the expression of CgCSAD2 was not in accordance with taurine. It may have little relationship with osmoregulation. In addition to being an organic osmolyte, taurine play a role in neuroregulation and calcium homeostasis in vertebrates’ studies^36, 37^. Remy, *et al*.^38^ found two copies of CSAD in brain and liver of mouse, respectively, and functions of them was similar. Although CSAD gene is the key enzyme in taurine biosynthesis pathway and the key to maintain cellular taurine balance, the two copies of CSAD in *C. gigas* have different expression patterns in response to low salinity. In oyster genome research, it is found that oyster stress responsive genes are more likely to retain paralogous duplications^20^. In this study, the results showed that the two copies of CSAD have different sub-functions. The specific function of CgCSAD2 in regulating taurine content needs further study. Comparisons between CgCSAD1 and CgCSAD2 in the molecular mechanism of functional specialization is of interest, and will advance the understanding of life evolution in adaptation to the variable environment.

## Electronic supplementary material

Figure S1
