# Supplementary material for: Identification and expression of cysteine sulfinate decarboxylase, possible regulation of taurine biosynthesis in Crassostrea gigas in response to low salinity
Source: Sci Rep. 2017 Jul 14;7:5505. doi: 10.1038/s41598-017-05852-6 (PMC5511178; doi:10.1038/s41598-017-05852-6)
Supplement: Supplementary file 1 — Figure S1 [file 41598_2017_5852_MOESM1_ESM.doc]

**Identification and expression of cysteine sulfinate decarboxylase, possible regulation of taurine biosynthesis in *Crassostrea gigas* in response to low salinity**

Xuelin Zhao­^1,2^, Qi Li^1,3^,*, Qian Meng^1^, Chenyang Yue^1^, Chengxun Xu^1^,

*^1^Key Laboratory of Mariculture, Ministry of Education, Ocean University of China, Qingdao 266003, China*

*^2^School of Marine Sciences, Ningbo University, Ningbo 315211, China*

*^3^Laboratory for Marine Fisheries Science and Food Production Processes, Qingdao National Laboratory for Marine Science and Technology*

^*^ Corresponding author: Q. Li, Fax: +86-532-82031765; E-mail address: qili66@ouc.edu.cn

Xuelin Zhao ([xlzhao1989@163.com](mailto:xlzhao1989@163.com))

Qi Li ([qili66@ouc.edu.cn](mailto:qili66@ouc.edu.cn))

Qian Meng (1508574120@.reureDW1 and WW1in family iliesal groups (WW00000000000000000000000000000000000000000000000000000000000000000000000000000000000qq.com)

Chenyang Yue ([yuechenyang1992@gmail.com](mailto:yuechenyang1992@gmail.com))

Chengxun Xu ([xcx_321@163.com](mailto:xcx_321@163.com))


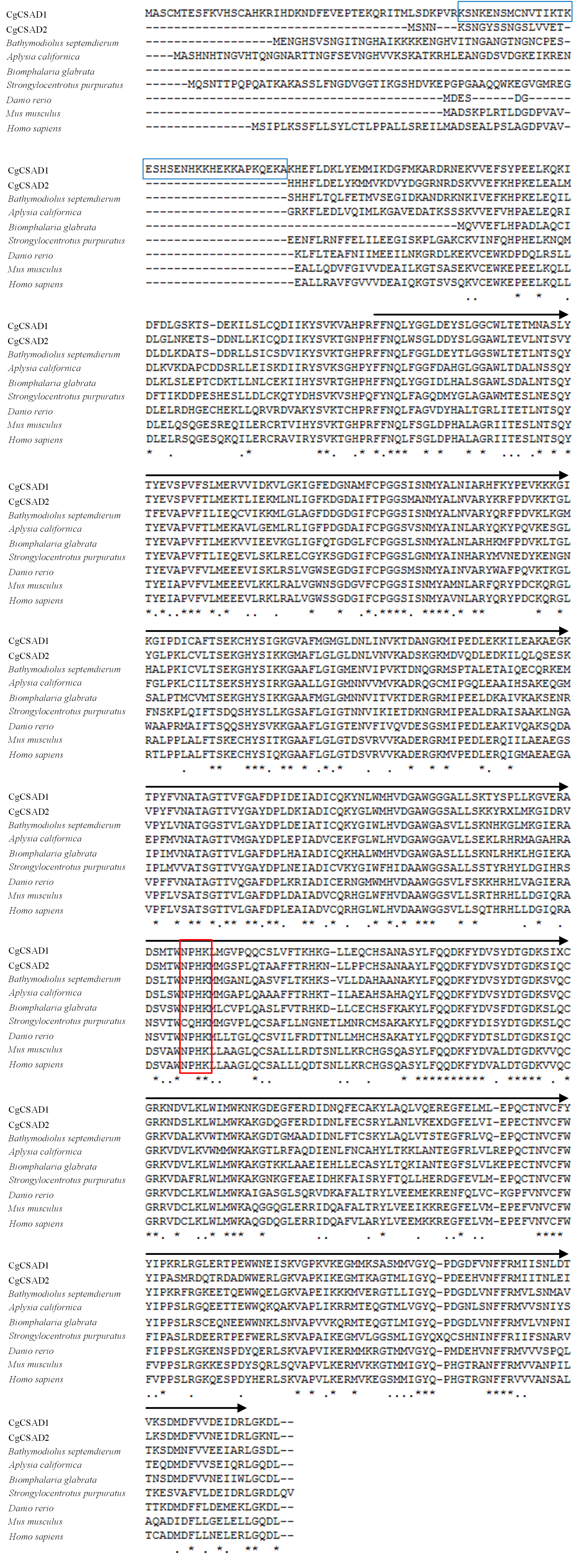


**Figure S1 Multi-alignment of CSAD between different species. The following species were included:** *Bathymodiolus septemdierum* CSAD (BAQ00225), *Aplysia californica* CSAD (XP_012935909), *Biomphalaria glabrata* CSAD (XP_013066301), *Strongylocentrotus purpuratus* CSAD (XP_011663410), *Danio rerio* CSAD (NP_001007349), *Mus musculus* CSAD (NP_659191), *Homo sapiens* CSAD (NP_057073). A blue rectangle indicates the ‘ZnF_GATA’ domain. The conserved PLP-dependent amino acid carboxylases domain are indicated in the top arrow. A red rectangle showed the conserved residues in PLP binding site.
